# Sec5 and Exo84 Mediate Distinct Aspects of RalA-Dependent Cell Polarization

**DOI:** 10.1371/journal.pone.0039602

**Published:** 2012-06-22

**Authors:** C. Clayton Hazelett, Charles Yeaman

**Affiliations:** Department of Anatomy and Cell Biology Carver College of Medicine, University of Iowa, Iowa City, Iowa, United States of America; Northwestern University Feinberg School of Medicine, United States of America

## Abstract

Metastasis is a complex process during which several gross cellular changes occur. Cells must dissociate from the tumor mass and gain the ability to degrade extracellular matrix and migrate in order to ultimately attach and form a satellite tumor. Regulation of the actin cytoskeleton is an indispensible aspect of cell migration, and many different factors have been implicated in this process. We identified interactions between RalA and its effectors in the Exocyst complex as directly necessary for migration and invasion of prostate cancer tumor cells. Blocking RalA-Exocyst binding caused significant morphological changes and defects in single and coordinated cell migration.

## Introduction

Cell migration and invasion are two indispensible functions necessary for metastasis to occur, and contribute to cancer mortality. In addition to mutations in tumor suppressors and oncogenes that promote cancer growth and tumor formation, cells also acquire increased motile capacity during metastasis. Loss of E-cadherin from cell-cell contacts is highly associated with tumor invasion [1], [2] and once cells gain the ability to invade surrounding matrix, the possibility of intravasation into nearby vasculature is likely increased. Therefore, a clear understanding of the processes that regulate tumor cell migration is an important subject of research, as such understanding may lead to advances in cancer treatment.

Since invasive cancer cells lose epithelial qualities, investigation of proteins involved with regulation of epithelial polarity may identify important factors involved in tumor cell migration. For example, Rho GTPases are involved with regulation of epithelial junctions and also cytoskeleton organization during cell migration [3], [4]. Furthermore, establishment of cell polarity is necessary during migration and many of the same proteins involved with initiation of epithelial polarization also regulate migration [5]. The Exocyst, an evolutionarily conserved hetero-octameric protein complex, has been implicated in each of these processes. Exocyst complex has been localized to lateral membranes [6] and developing apical domains of epithelial cells [7], [8], growth cones in neuronal cells [9], developing bud tips of growing yeast [10], in addition to being required for cell migration [11], [12]. Its functions are controlled by specific interactions with GTPases belonging to the Ras, Rho, Rab and Arf families.

Of the limited number of known Exocyst binding partners, much focus has been given to Ral GTPases and the cellular processes regulated by Ral-Exocyst interactions. RalA and RalB are closely related members of the Ras superfamily, and are activated by specific guanine exchange factors such as RalGDS family members [13]. GEFs of the Ral GDS family mediate many pro-metastatic functions of oncogenic Ras mutants [14]. Only four Ral effectors have been identified, and two (Sec5 and Exo84) are subunits of the Exocyst complex [15]. These effectors are known to bind competitively to Ral GTPases in a GTP-dependent manner and have been previously implicated in a number of processes relating to cell polarity including cell migration, tight junction formation, vesicle trafficking, and cytoskeleton regulation [11], [16], [17], [18].

In this study we investigated the role of RalA-Exocyst interactions in migration and invasion of tumor cells. We found that interactions between RalA and both Sec5 and Exo84 are required for single and coordinated cell migration and invasion through an underlying matrix. Certain characteristics of cell migration differed when specific Ral-Exocyst interactions were perturbed, and strikingly different phenotypes were observed upon abrogation of either interaction. Lastly, introduction of a compensatory Sec5 point mutation with ability to bind mutant RalA restores migration, invasiveness, and cell morphology.

## Results

### RalA-Exocyst Interactions are Necessary for Directed Cell Migration

We chose PC-3 cells, an invasive human prostate cancer cell line, to investigate the function of RalA-Exocyst interactions in tumor cell migration. To individually examine the roles of RalA-Sec5 and RalA-Exo84 binding, we introduced into PC-3 cells cDNAs with RalA point mutations that specifically and severely disrupt binding affinity for the Ral Binding Domain of Exocyst subunits Sec5 and Exo84 [19], [20]. The Sec5-uncoupled (E38R) and Exo84-uncoupled (K47E) mutations were produced in the context of an activating mutation, (Q72L). As a functional control, PC-3 cells stably expressing only the activating mutation were also generated. These point mutations are specific and do not affect interactions with other Exocyst effectors in cells, as RalA^38R^ and RalA^47E^ retain ability to bind Exo84 and Sec5, respectively, and neither mutation affects RalBP1 binding [16].

**Figure 1:**
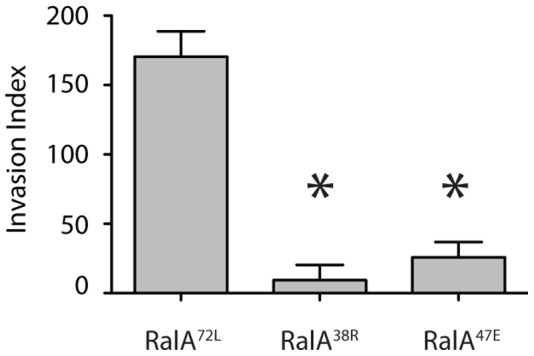
RalA-Exocyst interactions are required for Matrigel invasion. The invasion index of indicated cell types was determined and normalized to invasion of parental PC-3 cells. Invasion was determined by average # Matrigel invaded cells/average # migrated cells. Asterices, p<0.0001.

We first examined the effect of Exocyst-uncoupled RalA mutants on invasive ability by measuring invasion through an underlying Matrigel matrix. To differentiate between invasion and random cell migration, the average number of invaded cells was divided by the average number of cells that migrated across a non-coated Transwell filter to give a percent invasion, and normalized to parental PC-3 cells to identify an invasion index. RalA^72L^ cells displayed a high invasion index, as expected, while expression of either RalA^38R^ or RalA^47E^ significantly reduced this invasive ability (Figure 1). That expression of active RalA^72L^ had no significant effect on migration compared to parental PC-3 cells is consistent with a recent study showing that GTP hydrolysis is not required for effector dissociation [21].

**Figure 2:**
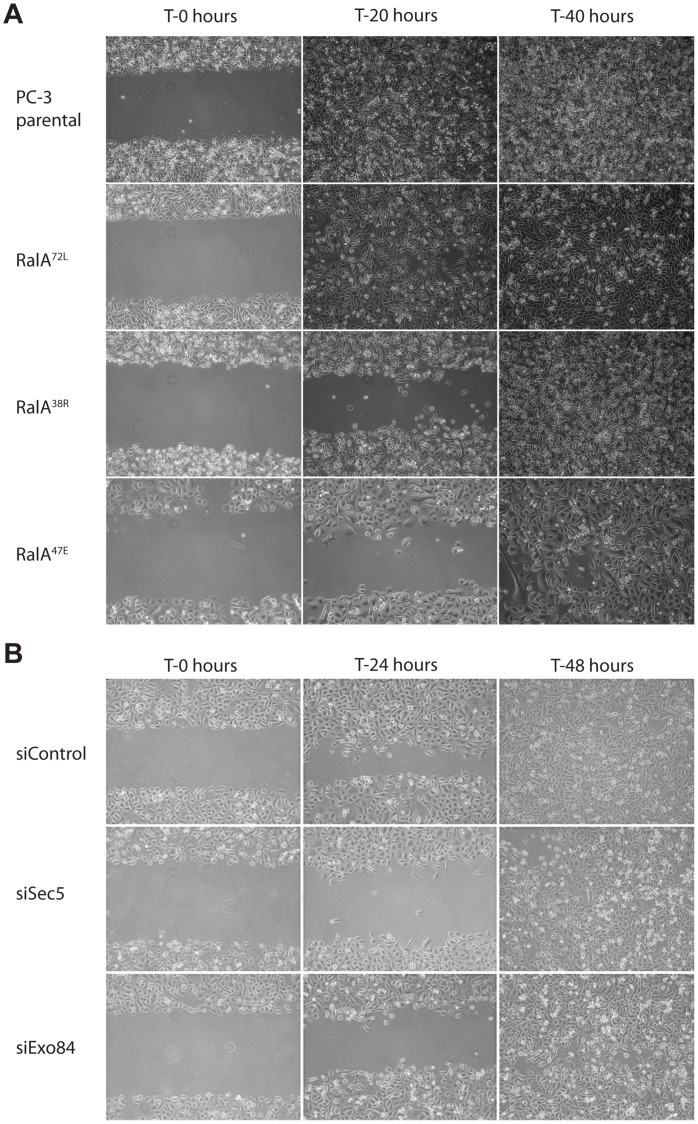
Coordinated cell movement is dependent on RalA-Exocyst interactions. Confluent monolayers of indicated cell types were scratched and images were collected either (A) 20 and 40 hours, or (B) 24 and 48 hours later.

Invasive ability of cells is dependent on both ability to migrate as well as ability to degrade extracellular matrix. To investigate whether RalA-Exocyst interactions are important for coordinated migration of cell populations, we performed *in vitro* scratch assays. After scratching confluent monolayers, parental and RalA^72L^ cells migrated to fill empty spaces within 20 hours, but RalA^38R^ and RalA^47E^ cells failed to completely close wounds at this time (Figure 2A). To further substantiate functions of RalA-Exocyst interactions in coordinated migration of PC-3 cells, we utilized siRNAs specific to Sec5 and Exo84 to reduce expression of each (Figure S1). Knockdown of Sec5 or Exo84 phenocopied observed effects of expressing Exocyst-uncoupled RalA mutants, as siSec5 and siExo84 treated cells were delayed in their ability to close wounds compared to siControl treated cells (Figure 2B).

**Figure 3:**
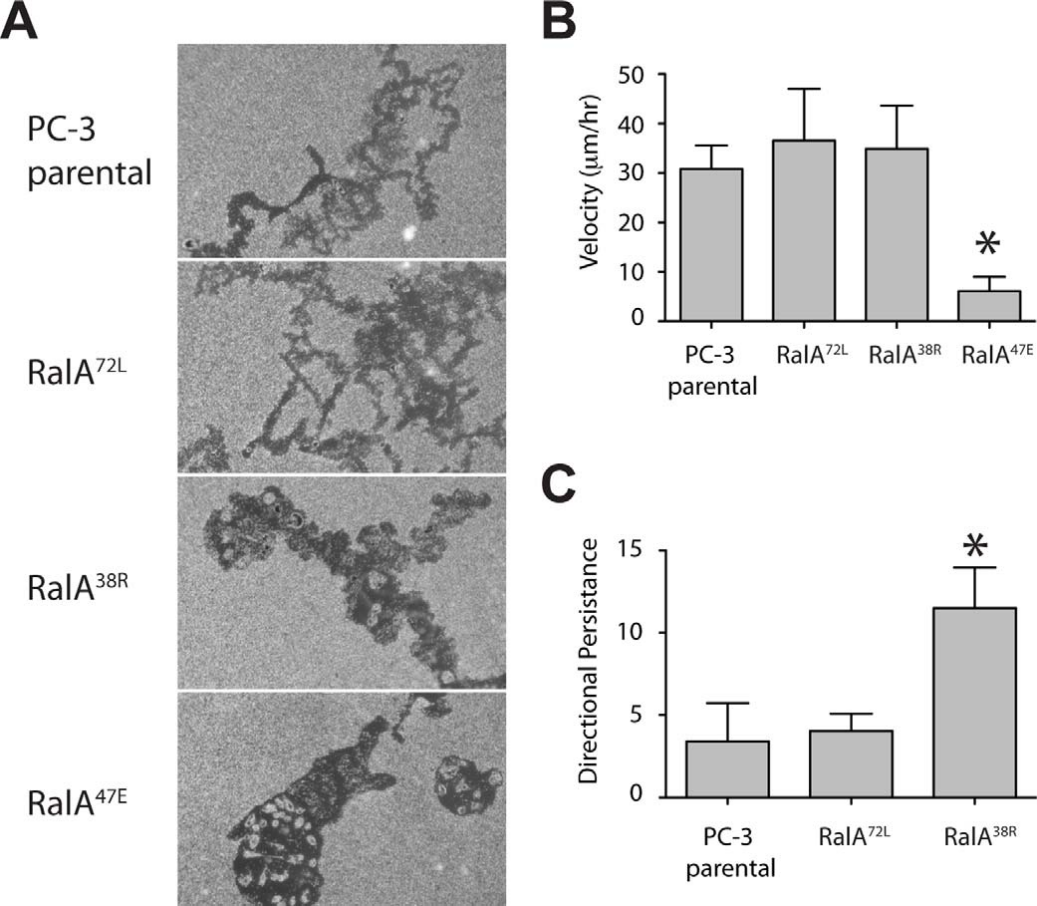
RalA-Sec5 and -Exo84 interactions differentially affect velocity and persistence of single cell migration. (A) Colloidal gold-coated coverslips were stained with crystal violet after allowing random migration of indicated cell types at low density. (B) Time-lapse photography was used to determine velocities of individual migrating cells. For each cell type, 5 cells from 4 fields of view were quantified during a 3-hour period. (C) From time-lapse photography images, distances between cell positions at the start and end of the in the 3-hour period were quantified and used to determine directional persistence. Directional persistence is defined as total distance migrated/net displacement. Asterices, p<0.0001.

Given the observed defects in Matrigel invasion and coordinated migration, we postulated that expression of Exocyst-uncoupled RalA mutants would also cause deficiencies in processes inherent to single cell migration. To visualize single cell migration, we initially performed phagokinetic track assays, using cells seeded at low density on colloidal gold-coated coverslips. Tracks of parental and RalA^72L^ cells were comparable, and displayed expected random motility (Figure 3A). RalA^38R^ and RalA^47E^ cells appeared to remain in a fairly concentrated area and formed very thick tracks, presumably from frequent changes of direction (Figure 3A). We next determined the migration velocity of each cell type using time-lapse photography and gridded coverslips. Parental, RalA^72L^ and RalA^38R^ cells all migrated at similar velocities, but RalA^47E^ average cell velocity was significantly reduced (Figure 3B). As a final measure of cell migration, directional persistence was calculated to quantify continued migration in a consistent direction. As with previous measures of cell motility, active RalA had no affect on persistence compared with parental cells, but RalA^38R^ cells were significantly less persistent (Figure 3C). Due to the loss of migration by RalA^47E^ cells, application of the persistence calculation was inappropriate for these cells. Thus, while the motility of RalA^47E^ cells was inhibited, that of RalA^38R^ cells was unaffected relative to controls. However, these cells changed direction often and lacked sustained migration in any single direction.

### RalA-Sec5 and RalA-Exo84 Uncoupled Mutants Differentially Affect Cell Morphology and Exocyst Localization

**Figure 4:**
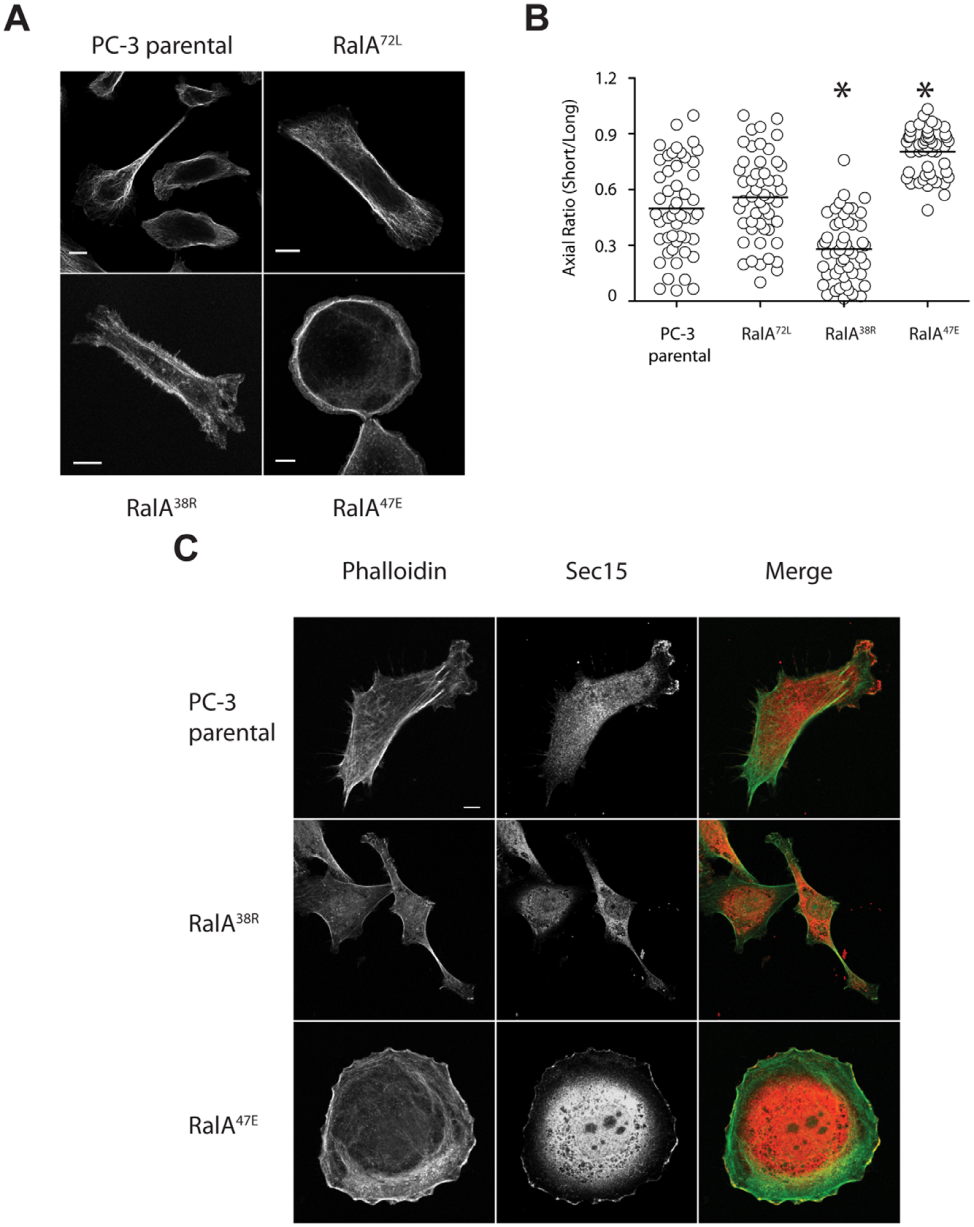
Cell morphology and Exocyst localization is differentially affected by uncoupling RalA from Sec5 or Exo84. (A) Indicated cell lines were labeled for phalloidin (f-actin). (B) The long and short axes of 50 cells of each type were measured and plotted. (C) Indicated cell lines were labeled with phalloidin (f-actin, green) and Sec15 (red). Representative images are shown. Bars, 10 µm. Asterices, p<0.0001.

We hypothesized that RalA-Exocyst interactions were required for basic processes inherently important for cell migration, and observed significant differences in overall cell morphology that could be responsible for these effects. Actin cytoskeleton labeling shows parental and RalA^72L^ cells with broad lamelliapodia at leading edges and trailing uropods, typical of migrating cells, while contrasting morphologies were observed in RalA^38R^ and RalA^47E^ cells (Figure 4A). RalA^38R^ cells displayed a much longer and extended spindle morphology in which no lamellipodia were observed in contrast to the circular shape of RalA^47E^ cells in which ruffled lamellipodia completely encircled cells. These morphologies were quantified by measuring short and long axes of each cell type to determine axial ratios. Average axial ratios of parental and RalA^72L^ cells were comparable at 0.5–0.6 while those of RalA^38R^ cells and RalA^47E^ cells were closer to 0.3 and 0.8, respectively (Figure 4B).

Since the Exocyst is intricately involved with several aspects of cell migration, we sought to determine its normal subcellular localization and whether this is affected by disruption of RalA binding to Sec5 or Exo84. Exocyst localization, as determined by Sec15 labeling, was concentrated at edges of parental PC-3 cells at lamellipodia. Notably, RalA^38R^ cells lost Sec15 labeling as well as distinct lamellipodia at leading edges. Conversely, lamellipodia extended most or all the way around RalA^47E^ cells, and Sec15 labeling was retained at these sites (Figure 4C). These data suggest that while both RalA-Sec5 and RalA-Exo84 binding are ultimately required for cell migration, each interaction may serve a distinct function during this process.

**Figure 5:**
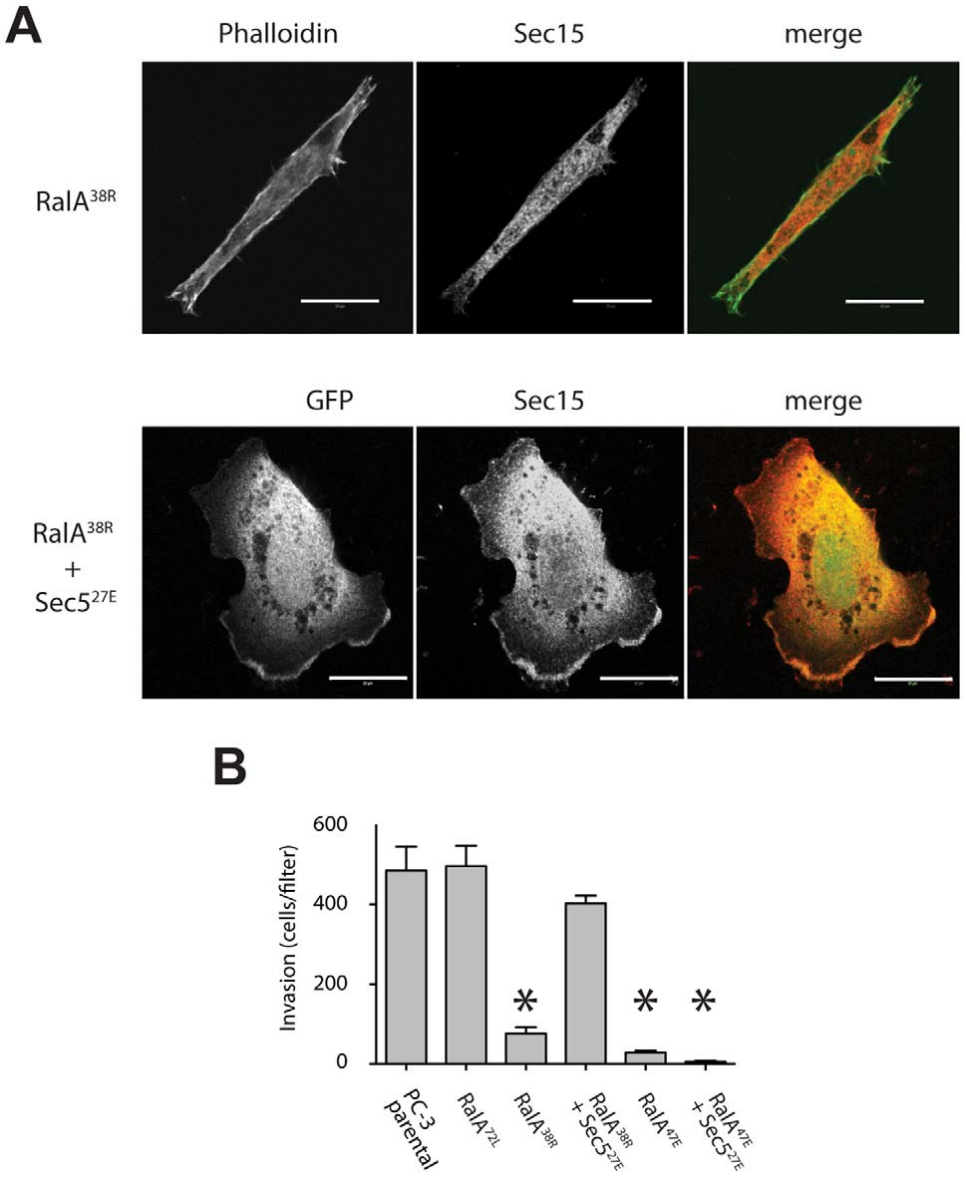
Mutant Sec5 capable of binding mutant RalA rescues morphological and functional defects. (A) RalA^38R^ cells were labeled with phalloidin (green) and Sec15 (red) and RalA^38R^ cells co-expressing GFP and Sec5^27E^ were labeled with Sec15 (red). (B) Matrigel invasion of RalA mutant cells alone and co-expressing Sec5^27E^. Bars, 20 µm. Asterices, p<0.0001.

To confirm that phenotypes associated with expression of mutant RalA^38R^ were due to a disruption of RalA-Sec5 binding, and not a heretofore unidentified interaction with a novel effector, we sought to determine whether expression of a complementary mutant Sec5 could rescue wild-type Exocyst localization, cell morphology, and invasive behavior. An amino acid substitution in Sec5 (R27E) restores binding to mutant RalA^38R^ [19]. Co-expression of Sec5^R27E^ in RalA^38R^ cells was sufficient to revert cell morphology from long and spindly to a phenotype more characteristic of parental PC-3 cells (Figure 5A). Furthermore, rescue of RalA-Sec5 binding restored Exocyst localization to lamellipodia. To determine whether this rescue was also functional, we again investigated invasion ability of control, RalA^72L^ cells, and RalA^38R^ cells and RalA^47E^ cells with or without Sec5^27E^. As expected, Sec5^27E^ expression rescued invasion of RalA^38R^ cells but did not affect invasion of RalA^47E^ cells (Figure 5B). This further demonstrated the specificity of the RalA^38R^ rescue.

### RalA-Exocyst Interactions Affect Regulation of the Actin Cytoskeleton

**Figure 6:**
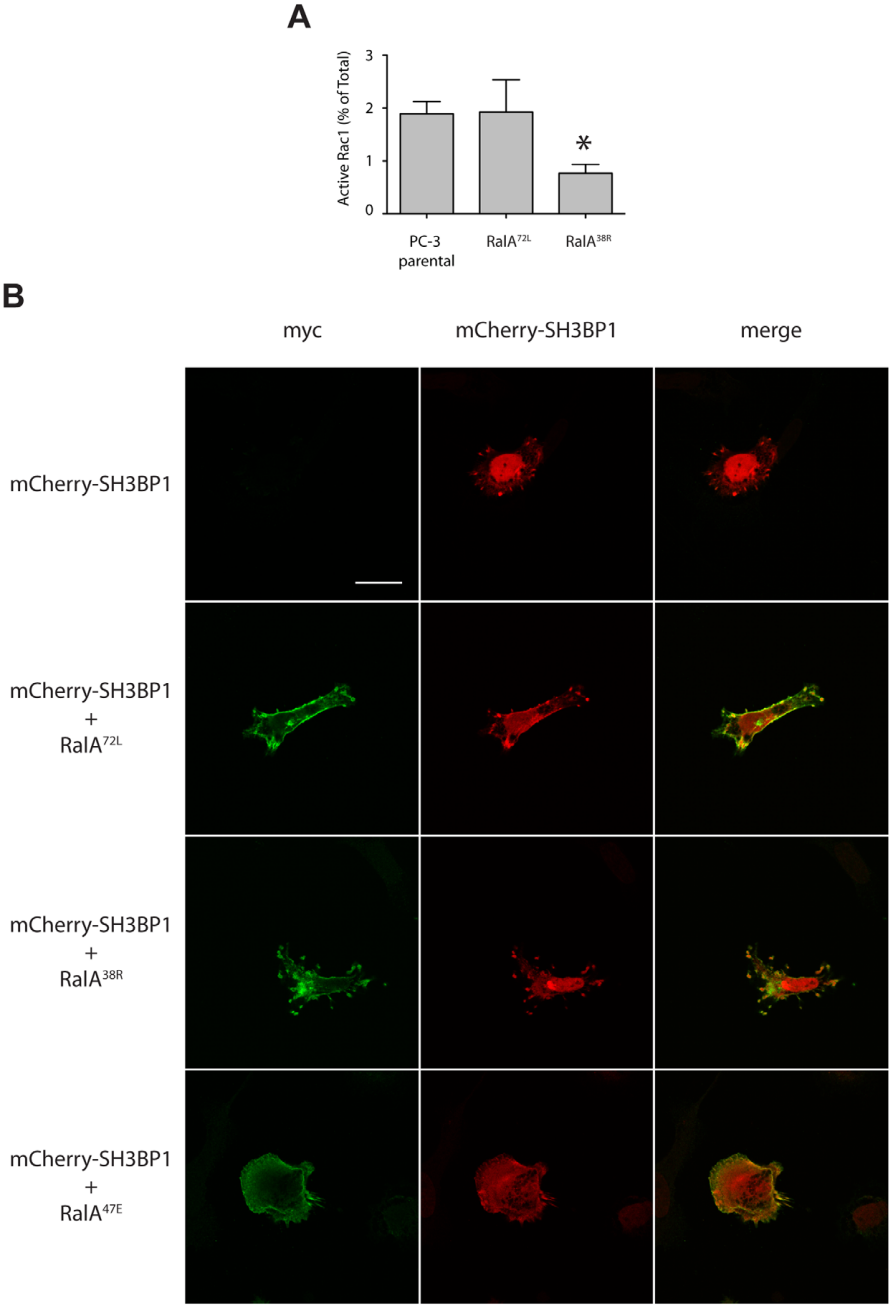
RalA-Exocyst interactions affect Rac1 activation through SH3BP1 localization. (A) Active Rac1 was isolated with GST-Pac binding domain bound to glutathione beads. Samples were visualized by western blot in triplicate and normalized to total Rac1 and expressed as percent of total Rac1. (B) PC-3 parental cells were transfected with mCherry-SH3BP1 only or together with myc-RalA^72L^, RalA^38R^, or RalA^47E^ and labeled with antibodies specific for myc. Asterisk, p<0.05. Bar, 20 µm.

Given the morphological defects resulting from uncoupling RalA-Exocyst interactions, we hypothesized that these interactions directly affect activation of cytoskeleton regulatory proteins. Specifically, we investigated Rac1 activation by performing GTP-pulldown assays using a GST-Pac binding domain. Densitometry analysis of western blots determined that the percent of active Rac1 was similar in PC-3 parental and RalA^72L^ cells, but was significantly decreased in RalA^38R^ cells (Figure S2 and Figure 6A). Thus, loss of RalA-Sec5 interactions causes reduced Rac1 activation and provides insight into the observed loss of lamellipodia in RalA^38R^ cells.

Rac1 activity can be indirectly affected through many different pathways in response to a variety of cell signals, but guanine exchange factors (GEFs) and activating proteins (GAPs) are directly responsible for activating or inactivating these proteins. SH3BP1 is a Rac1 GAP that associates with the Exocyst and is required for cell migration [22]. We transiently co-expressed active and Exocyst-uncoupled RalA with mCherry-fused SH3BP1 to determine whether SH3BP1 localization was dependent on RalA-Exocyst interactions. mCherry-SH3BP1 localized to similar structures at the cell periphery in cells expressing endogenous RalA, RalA^72L^, and RalA^38R^ (Figure 6B). Expression of RalA^47E^ shifted mCherry-SH3BP1 distribution from a tightly concentrated plasma membrane-associated pool to a more diffused one throughout the cell. Reduced SH3BP1 localization at lamellipodia in RalA^47E^ cells would promote elevated active Rac1 levels at these sites, and could account for the exaggerated lamellipodia observed in these cells.

## Discussion

This study has found that loss of RalA-Exocyst interactions causes functional defects that decrease the migratory and invasive abilities of PC-3 cells. Specifically, RalA-Sec5 and -Exo84 uncoupled cells displayed reduced Matrigel invasion and coordinated cell migration, as well as decreased velocity and directional persistence (Figures 1–3). Furthermore, different cell morphologies were observed in response to uncoupling RalA from Sec5 or Exo84. Sec5-uncoupled cells lost lamellipodia and developed an extended spindle shape while Exo84-uncoupled cells formed exaggerated lamellipodia and became significantly rounder (Figure 4). Lastly, Exocyst localization to lamellipodia was differentially affected by RalA-Exocyst interactions, and expression of Sec5^27E^ was sufficient to rescue loss of lamellipodia and Exocyst localization in RalA^38R^ cells (Figures 4 and 5). These functional defects in migration and invasion naturally follow from the morphological differences that result from specifically disrupting RalA-Exocyst interactions.

Although each Exocyst-uncoupled RalA mutant promotes impaired cell migration when expressed in cells, the basis for these defects is most likely due to differential impacts on cell polarity. Initially, it could be reasoned that since RalA^47E^ cells form exaggerated lamellipodia, they would migrate and invade more than parental PC3 cells. However, the extent of lamellipodia is such that the leading edge extends, in some cases, entirely around the cell. This would significantly reduce migration ability, as cells would be pulled in multiple directions simultaneously causing virtually no migration. The reduced velocity of these cells is also apparent in coordinated cell migration, and to a greater extent in the more complex process of Matrigel invasion. In contrast, RalA^38R^ cells migrate at speeds similar to control PC-3 cells but nevertheless exhibit reduced invasiveness and coordinated cell migration. This can be explained by reduced directional persistence and by lack of sustained lamellipodia. Without lamellipodial leading edges, these cells change direction significantly more frequently and continue in one direction for less time than control cells. Lack of a phenotype and retention of Sec5 and Exo84 binding by RalA^72L^ cells provides insight to potential methods of migration regulation by Ral-Exocyst interactions. A straightforward and testable hypothesis that stems from these results is that sequential engagement of Sec5 and Exo84 by Ral GTPases is required to complete opposing functions necessary for cell migration. We suggest that RalA binding to Sec5 “activates” the Exocyst, and that subsequent binding of RalA to Exo84 “inactivates” the Exocyst for such functions. Thus expression of RalA mutants and resulting interruption of either interaction would alter the balance of Ral-Exocyst complexes, but RalA^72L^ expressing cells maintains this equilibrium.

The Exocyst has been previously described as a scaffold, serving to localize various factors necessary for specific cellular processes [12], [22], [23]. We propose that this process occurs during tumor cell migration and invasion, and that Exocyst-association and localization of factors is allosterically regulated by RalA interactions with both Sec5 and Exo84. We found that Rac1 activation is significantly decreased in RalA^38R^ cells, which is supported by the morphological phenotype of reduced lamellipodia (Figures 4 and 6). This suggests that RalA-Sec5 interactions are necessary for Rac1 activation and formation of lamellipodia. Furthermore, loss of SH3BP1 localization at the cell periphery in RalA^47E^ cells suggests that RalA-Exo84 interactions are also necessary to properly regulate lamellipodia formation. Our data thus support a situation in which RalA-Sec5 binding could recruit and localize GEFs involved in lamellipodia formation, and RalA-Exo84 binding could function to localize GAPs necessary for restriction of lamellipodia to leading edges.

In summary, this study has identified a role for RalA-Exocyst interactions in migration and invasion of highly motile PC-3 cells. Furthermore, these interactions may mediate cytoskeleton remodeling events required for the invasive properties of these cells. Additional work will be necessary to determine the mechanism by which RalA-Exocyst interactions affect the morphological differences observed here, and whether these functions occur in other cell types.

## Materials and Methods

### Cell Culture

PC-3 cells were maintained in DMEM/F12 supplemented with Dulbecco’s Modified Eagle Medium Nutrient Mixture F-12 (Ham) [DMEM/F-12 (1∶1); Gibco, Invitrogen, Carlsbad, CA] supplemented with MEM Non-Essential Amino Acids (MEM-NEAA; Gibco), 10% fetal bovine serum (FBS; Hyclone) and penicillin, streptomycin, and gentamicin (PSG) and grown at 37°C with 5% CO_2_. PC-3 cells stably expressing rat RalA^72L^, RalA^72L38R^, or RalA^72L47E^ were generated by stable integration of RalA by co-transfection of each pmT3myc-RalA construct with pmv NEO using Lipofectamine per manufacturer’s instructions. Cells were selected with 300 µg/ml G418. For Sec5 and Exo84 knockdown experiments, PC-3 cells were nucleofected with control, Sec5, and Exo84 siRNAs (5′-GAAATTGCACATTCACAGC-3′, 5′-ACGGCAGAATGGATGTCTGC-3′, 5′-AAGGTGCCACTTTACTCTATA-3′, respectively; Dharmacon, Lafayette, CO) using the Amaxa system (Lonza Group Ltd, Switzerland) with Solution T and Program T-020 according to manufacturer’s instructions. For RalA-Sec5 rescue experiments, PC-3 cells were transiently co-transfected with pEGFP and pCMV-myc Sec5^R27E^ cDNA carrying a point mutation that enables binding to RalA^72L38R^ following the same methods used for siRNA nucleofection or with Lipofectamine 2000 (Invitrogen, Carlsbad, CA), following manufacturer’s instructions.

### Immunofluorescence

Samples were seeded onto glass coverslips coated with rat tail collagen and fixed on ice with 4% paraformaldehyde for 20 minutes prior to quenching with Ringer’s saline (154 mM NaCl, 1.8 mM Ca^2+^, 7.2 mM KCl, and 10 mM HEPES, pH 7.4) containing 50 mM NH_4_Cl. Samples were then permeabilized with CSK buffer (1% TritonX-100, 10 mM Pipes, pH 6.8, 50 mM NaCl, 300 mM sucrose, 3 mM MgCl_2_) containing protease inhibitors (1 mM pefabloc and 10 µg/ml each of aprotinin, antipain, leupeptin, and pepstatin A) for 10 minutes. Samples were blocked with 0.2% fish-skin gelatin in Ringer’s saline (blocking buffer) for 1 hour and primary antibodies diluted in blocking buffer were applied for 1 hour. After 5 washes with blocking buffer, FITC or Texas Red-conjugated secondary antibodies and DAPI were applied for 30 minutes. Samples were washed 5 times with blocking buffer and coverslips were mounted onto slides using Elvanol-PPD. Images were obtained using a Zeiss 510 scanning confocal microscope (Thornwood, NY; 63X objective) equipped with a krypton/argon laser (FITC excitation using 488 nm laser line, or and Texas Red excitation using 543 laser line).

### Antibodies and Reagents

Mouse monoclonal antibodies against Sec5 and Exo84 were generously provided by Dr Richard Scheller (Genentech). Rabbit polyclonal antibodies against Sec15 were generated by Covance and have been described previously. Fluorescein isothiocyanate (FITC)-goat anti-mouse, Texas Red (TR)-donkey anti-rabbit immunoglobulin (Ig)G, and Fluorescein isothiocyanate (FITC)-goat anti-phalloidin were purchased from Jackson ImmunoResearch Laboratories (West Grove, PA). Horseradish peroxidase-conjugated goat anti-mouse and goat anti-rabbit antibodies were purchased from Promega (Madison, WI). Matrigel was purchased from BD Biosciences (Bedford, MA) and mouse-anti myc clone 4A6 antibodies were purchased from Millipore (Billerica, MA). Mouse-anti Rac1 antibodies were purchased from BD Transduction Laboratories; San Jose, CA). mCherry-SH3BP1 was a generous gift from Dr. Jacques Camonis (Institut Curie, Paris France).

### Invasion Assay

Each cell type was trypsinized, centrifuged and resuspended in Opti-Mem (Gibco) supplemented with 0.5% BSA to yield a density of 5×10^5^ cells/ml. 100 ul cell suspension was seeded on 6.5 mm Transwell® filters either uncoated or coated with 20 ul Matrigel for 2 hours at 37°C. Following initial attachment, media in basal champers was replaced with DMEM containing 10% FBS and filters were incubated for 24 hours at 37°C. Filters were washed with Ringer’s and apical surfaces of filters were wiped with a cotton swab to remove all cells. Apical chambers were washed twice again, fixed and quenched as described for immunofluorescence, and mounted on slides with VECTASHIELD mounting medium with DAPI (Vectashield Laboratories, CA). The nuclei from 15 fields of view on the bottom of 3 filters per cell type were counted. Cells on uncoated filters represent “migrated cells” and cells on Matrigel coated filters represent “Matrigel invaded cells”. Invasion index of each cell type was determined by average # Matrigel invaded cells/average # migrated cells to give % invasion, which was normalized to parental PC-3 cells to give invasion index (which have an invasion index of 100). Significant differences were determined by one-way analysis of variance with Tukey’s Post test; asterisks, p<.0001.

### Wound Healing

Cells were seeded in 6-well chambered tissue culture plates and grown until confluent. All media was changed prior to scratching the monolayer with a yellow pipette tip to create a wound of equal width in all cell types. Images were taken immediately after wounding and either 20 or 24 and 40 or 48 hours later.

### Phagokinetic Track Assay

Cell migration was investigated using a colloidal gold assay, which has been previously described in detail [24]. Briefly, colloidal gold suspension (11 ml ddH20+6 ml 36.5 mM Na2CO3+1.8 ml 19.5 mM AuCl4H) was brought to a boil, and 1.8 ml 0.1% paraformaldehyde was added and the solution simmered for 15 minutes. During this time, coverslips were coated with 1% BSA and subsequently dried. Coverslips were then incubated with colloidal gold suspension for 45 minutes before rinsing 3 times with DMEM. Cells were seeded on these coverslips at low density and grown for 4 days. On ice, coverslips were fixed and quenched as described for immunofluorescence. Coverslips were then counterstained with 0.5% crystal violet for 10 minutes and washed repeatedly with Ringer’s until all excess dye was removed. Coverslips were then post-fixed with 3.7% formaldehyde in Ringer’s for 20 minutes, washed with Ringer’s, and mounted on slides with Elvanol. Images were obtained using a TE300 microscope (Nikon) equipped with a Coolpix 5000 digital camera (Nikon).

### Velocity and Persistence

Cells were seeded at low density on gridded coverslips and images were obtained every 20 minutes for 3 hours total. 5 individual cells in 4 different fields of view per cell type were tracked and velocities (µm/hr) determined. Directional persistence was defined as total distance migrated/net displacement, where displacement is the distance between the starting and ending positions of each cell.

### Axial Ratio

For 50 cells of each cell type, the long axis and the width at the midpoint of the long axis were measured and the ratio determined. One-way analysis of variance with Tukey’s Post Test was applied to determine statistical differences. Asterisks, p<.0001.

### Rac1 Pulldown

GST-Pac fusion protein was pre-bound to glutathione sepharose beads, and indicated cell lines grown to 80% confluence were used. The same methods were used as described previously [16], except cells were not serum starved in this case. Samples were subjected to western blotting and probed with antibodies specific to Rac1.

## Supporting Information

Figure S1 **Transient knockdown of Sec5 and Exo84 in PC-3 cells.** Western blot of Sec5 and Exo84 in control and si- treated cells indicated specific knockdown of Sec5 and Exo84.
(TIF)

Figure S2 **Rac1 activation is affected by RalA-Exocyst interactions.** Western blot of active Rac1 isolated from indicated cell types. Total lysates and pulldowns are shown.
(TIF)
