# Supplementary figures and images for: Sec5 and Exo84 Mediate Distinct Aspects of RalA-Dependent Cell Polarization
Source: PLoS One. 2012 Jun 22;7(6):e39602. doi: 10.1371/journal.pone.0039602 (PMC3382198; doi:10.1371/journal.pone.0039602)

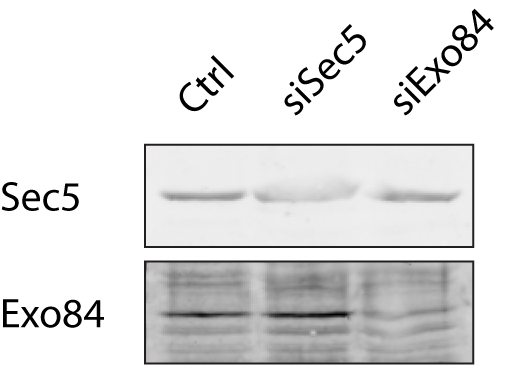

Supplement: Figure S1 — Transient knockdown of Sec5 and Exo84 in PC-3 cells. Western blot of Sec5 and Exo84 in control and si- treated cells indicated specific knockdown of Sec5 and Exo84. (TIF) [file pone.0039602.s001.tif]

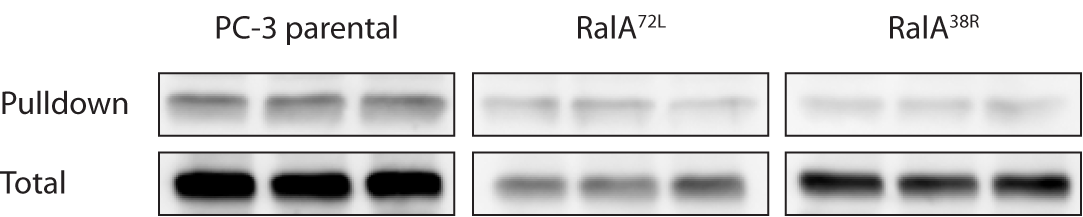

Supplement: Figure S2 — Rac1 activation is affected by RalA-Exocyst interactions. Western blot of active Rac1 isolated from indicated cell types. Total lysates and pulldowns are shown. (TIF) [file pone.0039602.s002.tif]
